# Enhancement of humoral immunity in mice by coupling pUCpGs10 and aluminium to the HCV recombinant immunogen

**DOI:** 10.1186/1743-422X-8-507

**Published:** 2011-11-04

**Authors:** Na Zhan, Bing S Xiu, Guo H Wang, Kun Chen, Guan Z Bai, Xiao G Song, Cui X Zhu, Zhen H Dai, Xi Q Yang, He Q Zhang

**Affiliations:** 1 Institute of Basic Medical Sciences, Academy of Military Medical Sciences, Taiping Road No.27, Haidian, Beijing 100850, China; 2 Hospital 307 of PLA, Academy of Military Medical Sciences, Fengtai, Dongda Road No.7, Beijing 100072, China

**Keywords:** HCV, humoral immunity, adjuvant, ELISPOT, FCM

## Abstract

**Aim:**

To investigate the enhancement of humoral immunity when CpG ODN (cytidine phosphate guanosine oligodeoxynucleotides) and aluminium adjuvants are complexed with the HCV (Hepatitis C virus) recombinant immunogen in mice.

**Methods:**

After immunizing Balb/c mice with the recombination HCV antigen adjuvanted with pUCpGs10 and/or aluminium(antigen+CpG+alum, antigen+CpG, antigen+alum, antigen+PBS), enzyme-linked immunosorbent assay (ELISA) was used to measure the specific serum antibody titers of IgG, to determine the neutralization response to various peptide genotypes, and to determine the concentration of IL-6 and IL-10 in supernatants of in vitro cultured splenic lymphocytes. Enzyme-linked immunospot assay (ELISPOT) was used to quantify the non-specific and specific splenic antibody-secreting cells (ASCs), and flow cytometry (FCM) determined the ratio of different splenic lymphocytes. The serum of rabbits immunized with the recombinant pBVGST/HVR1 antigen immunoprecipitated the HCV isolated from 12 patients' serum.

**Results:**

The sera antibody titers were 1:51200, 1:9051, 1:18102, 1:6400 respectively after the final immunization and demonstrated good neutralization responses to the six gene peptide containing 1a, 1b, 2a, 3a, 4a and 6a. The aluminum adjuvant increased the population of both specific ASCs (P < 0.01) and total ASCs(P < 0.05), with a proportional rise in concentrations of CD19^+^CD27^+ ^(P < 0.05), as well as levels of IL-6, IL-10 (P < 0.05) in splenic lymphocytes. The results clearly indicated a significantly higher number of CD19^+^CD38^+ ^splenic lymphocytes with the aluminum and pUCpGs10 adjuvant present compared to the control group(P < 0.05). Anti-HVR1 antibody in induced mice can cross-reactively capture HCV particles (10/12).

**Conclusions:**

1. The aluminum adjuvant induces a potent Th2-biased immune response by increasing both the populations of specific and total ASCs and the ratio of CD19^+^CD27^+ ^cells. 2. The pUCpGs10 complexed with the aluminum adjuvant boosts the population of plasma cells and increase the efficiency of the immune response. 3. The two adjuvants have synergistic effects on humoral immunity. 4. The recombinant HVR1 protein has the possibility of generating broadly reactive anti-HVR1 antibody.

## 1. Introduction

At present, more than 200 million people worldwide are infected with HCV [1], and are therefore at risk of developing liver cirrhosis and hepatocellular carcinoma. HCV has been shown to impair the humoral immunity response in several ways [2,3]. For example, HCV can induce resistance of infected hepatocytes to type I IFNs and HCV E2 inhibits NK cells. Viruses escape from immune responses through mutation in antibody and T cell epitopes has been shown for both HCV-infected humans and chimpanzees. In addition, potential mechanisms include reduced T-cell priming with a potentially altered DC(dentritic cell) function and inhibition of macrophage, DC and T-cell function through binding of the HCV core protein to the receptor for the complement component C1q(C1qR).

The constant changes that occur to HCV variants make it difficult to neutralize the virus and develop vaccines based on a single specific antibody. However, an effective vaccine enhances host humoral immune responses in an antigen-specific manner by producing a broader spectrum neutralization antibody. Various peptides containing the B and T cell epitopes have been synthesized, such as recombinant polyprotein HVR1 and E1(HVR1: VARAAFGLTSIFSPGAKQN, GTHVTGGKVAYTTQGFTSFFSRGPSQK, QTTVVGGSQSHTVRGLTSLFSPGASQN, TTHTVGGSVARQVSHLTGLFSPGPQQKGSASSSEGGSTTTTTGGVQGHTTRGLVRLFSLGSKQN; E1: YQVRNSSGLYHVTNDCPNSS, YEVRNVSGVYHVTNDCSNSS, VQVKNTSSSYMVTNDCSNDS, LEWRNTSGLYVLTNDCSNSS, VHYRNASGVYHVTNDCPNTS, LTYGNSSGLYHLTND CPNSS.) involving different genotypes and variations of the quasi-species which conclude 6 kinds of genotype and the response rate to the sera of the HCV infected patients is more than 90% [4,5]. In order to obtain higher titers of the antibody to the polyprotein, adjuvants are essential. Adjuvants augment the immunological response of an organism by enhancing humoral immunity in different ways [6]. There has been study about cellular mechanism of coupling CpG and aluminum to HBV instead of humoral mechanism to HCV [7].

### 1.1. pUCpGs10

When CpG ODN is applied as the adjuvant of the HCV vaccine it significantly stimulates innate immunity by specifically binding pDC TLR9 to B lymphocyte [8,9], as is the agonist of the toll-like receptor 9(TLR9). CpG ODN has great potential to be used as a vaccine adjuvant or a modulator of immunotherapy. For example, TLR9 signals can regulate B lymphopoiesis *in vivo *[10]. pUCpGs10 which is fabricated by the institute of Basic Medical Sciences (patent No.200710110466.7)containing eleven motifs of CpG inserts repeating ten times in the pUC19 vector, which was invented by this research group, and directly activates signal transduction causing cell division and cytokine secretion. pUCpGs10 shows adjuvant activity towards almost all of the protein antigens and inactivated vaccines. The main contributions of CpG ODN include the promotion of the cytokine secretion(IFN-α/β, γ)and anti-virus reaction, increases in NK cell and macrophage cytotoxicity, enhancement of antibody titer, elevation of the expression of MHC and immune cofactors, and increase the Th1 cellular immunologic response to antigenic specificity [11]. Mouse B cells express a number of different toll-like receptors (TLRs) including TLR3, TLR4, TLR7 and TLR9. The stimulation of mature B cells with TLR ligands induces B cell activation, proliferation and differentiation into antibody secreting cells [12-15].

### 1.2. Aluminum

Aluminum hydroxide is the only inorganic adjuvant currently in use. It is approved by the US FDA for vaccine formulation and has a very good safety profile. Any adverse reaction to aluminum adjuvant is not clinically significant and is localized to the injection site [16,17]. Aluminum induces the Th2 immune response in animal models, stimulates proliferation of T cells *in vitro*, promotes differentiation of histoleucocytes into mature CD83^+^DCs, and proliferation of macrophages originating from bone marrow *in vivo*[18]. Aluminum hydroxide is positively charged at pH 7.4 and easily absorbs onto negatively charged antigen [19]. The regional accumulation combined with delayed release stimulates an effective and sustained B lymphocyte immune reaction [20,21].

### 1.3. Specific antibody-secreting cell

Naïve B cell contacted antigen with BCR (B cell receptor), then it proliferated into the antigen specific B cell. The specific B lymphocytes ultimately differentiate into plasma and memory B cells in the germinal center. In the second immune response, memory B cells absorb and present antigen as APC to the memory Th cell. The activated Th cells express multiple membrane molecules and show enhanced secretion of cytokines, which induces the memory B cells to quickly proliferate and differentiate into plasma cells, that actively biosynthesize and secrete antibodies [22].

The current consensus is that BCR interacts with T cell to help promote B cell differentiation and proliferation to the antigen-specific B cell, and then on to the memory B cells or plasma cells. Through affinity maturation, plasma cells ultimately differentiate into antibody secreting cells (ASCs) [23]. Memory B cells can be activated, amplified, and selectively transformed into effector cells. In contrast to the poorly expressed TLR9 naïve B cells, the memory B cells constitutively express TLR9 and differentiate into immunoglobulin secreting cells when stimulated with CpG ODN [24].

The memory B cells can quickly differentiate and proliferate at any time during the secondary immune response. Unlike naïve B cells, the activation of memory B cells required a stimulation signal not only from Th but also BCR. The quantity of peripheral blood lymphocytes in patients persistently infected with HCV is reduced compared to healthy individuals and those who spontaneously recover from HCV infection. These differences may result from the weakening of post-stimulus humoral immunity and reduced quantity of specific ASC *in vitro *[25].

### 1.4. CD19^+^CD27^+ ^and CD19^+^CD38^+ ^cells of mice

CD19 is an important molecule within the B lymphocyte antigen receptor (BCR) complex that regulates signal transduction [26]. The early pro-B cells of mice express CD27^+^AA_4.1_^+^1/^-^Ki-67^+ ^Ly-6C^- ^Ly-6A/Sca-1^lo^/^-^Thy-1^-^2CD43^+^CD4^+^/^-^2CD16/32^lo/- ^and CD44^Hi^. Recent findings show that expression of CD27 permits the distinction between antigen-inexperienced naïve B cells and antigen-experienced memory B cells [27]. It is known that the frequencies of CD27^+^B cells in persistently infected patients are reduced and that CD27 ligation inhibits terminal differentiation of murine B cells into Ig-secreting plasma cells [24]. CD19 is the main marker of murine B cells and antibodies to mouse CD38 activate B lymphocytes in adult mice [28,29]. Both naive and memory B cells progress to a plasma-cell phenotype:CD19^low^CD_20_^low^CD27^+^CD38^+^HLA-DR^low ^[30]. CD38 is a marker for human bone marrow plasma cells and has been used extensively to monitor *in vitro *generation of ASCs from memory B cells. Crosslinking of CD38 not only induces the proliferation of mature follicular B cells but also enhances the proliferation and differentiation of the immature transitional 2 (T2) B cells. Biological outcomes after crosslinking CD38 on immature and mature mouse B cells are dependent on expression of a functional BCR [31]. The crosslinking of CD38 in mature B cells can be induced by toll-like receptor (TLR) signaling. Likewise, crosslinking of mature mouse B cells with anti-CD38 in the presence of cytokines such as IL-4 induces proliferation [27], while activation of B cells with anti-CD38 in the presence of IL-5 induces differentiation of B cells into IgG1-secreting plasma cells [32]. Specifically, CD38 catalyzes an ADP-ribosyl cyclase reaction, and likely plays an important role in inflammatory responses [33]. It is well known that foreign CpG-DNA from viruses and bacteria can activate memory B cells through binding to TLR9, and that this pathway may be involved in the continuous activation of memory B cells ensuring life-long humoral immunity [34]. In this study, the quantitative ELISPOT method for simultaneous estimation of single-cell IgG secretion rates and secreting cell frequencies in human B cell populations was used. These results suggest that CD27^+^IgM^_ ^memory B cells activated with CpG and cytokines exhibit considerable heterogeneity in IgG secretion rates, and two major secreting subpopulations were identified. BCR cross-linking reduced the frequency of cells with high per-cell IgG secretion rates, and this was associated with a parallel decrease in CD27^high ^B cell blasts. Increased cell death may account for the BCR-stimulated reduction in high-rate IgG-SC CD27^high ^B cell blasts [35].

## 2. Methods

### 2.1. Purification of antigen and immunization of Balb/c mice and rabbits

Activation of the genetically engineered bacteria, expression and purification of the HCV recombinated antigens pBVIL1/E1 and HVR1 were undertaken as previously described [3,4].

Female Balb/c mice, aged 6-9 weeks were obtained from the experimental animal center of the Academy of Military Medical Sciences (Beijing, China). Rabbit (New Zealand, weight: 3 kg) were obtained from KeYu breed factory of Beijing. The animals were housed and manipulated according to the Care and Use of Laboratory Animals (China), and kept under pathogen-free conditions.

The mice were randomly assigned into four groups and injected s.c. with 50 μg E1 and 50 μg HVR1 +100 μl colloidal(Al(OH)_3_,1:1, v/v; Therma Scientific) together with 20 μg pUCpGs10, 50 μg E1 and 50 μg HVR1 +20 μg pUCpGs10, 50 μg E1 and 50 μg HVR1+100 μl AL(OH) _3_, 50 μg E1 and 50 μg HVR1 formulated in PBS. The rabbits were injected (s.c.) with 100 μg HVR1 +110 μl colloidal(Al(OH)3,1:1, v/v; Therma Scientific) together with 20 μg pUCpGs10.

An interval of 4 weeks was employed for the second and third immunizations. All mice and rabbits were exsanguinated 2 weeks after the last immunization. Mice spleens were removed and splenocytes were analyzed using the enzyme-linked immunospot (ELISPOT) assay and the flow cytometry (FCM). Serum samples at 2, 6 or 10 weeks after immunization of mice and rabbits were tested for specific antibody titers.

### 2.2. ELISA assay

Sera were collected two weeks after the last immunization from vaccinated mice. IgG antibody titers in serum samples were assessed using a standard ELISA protocol. Briefly, microtiter plates were coated overnight at 4°C with 5 μg pBVIL1/E1 or HVR1 and synthesized peptidesE1a, E1b, E2a, E3a, E4a and E6a were added in 100 μl of 50 mM sodium carbonate buffer (pH 9.6) per well. The plates were washed thoroughly with PBS containing 0.05% of Tween-20 (PBST) and then blocked with 110 μl of PBS containing 20% goat serum albumin for 8 h at room temperature. Two-fold serially diluted serum samples were allowed to react with coated plates at 37°C for 30 min, followed by 0.05% PBST washes. They were then incubated with a 1:10000 dilution of horseradish peroxidase conjugated goat anti-mouse IgG antibody (Bio-Rad) at 37°C for 20 min. After washing the plates, 100 μl of TMB was added to each well to allow colour development at room temperature for 10 min before the reaction was stopped by adding 50 μl of 2M H_2_SO_4_. Absorbance was measured at 450 nm using an IEMS reader. Antibody titers were expressed as the reciprocal of the last sample dilution giving an absorbance of at least two-fold that of the pre-immune sample and with an OD ≥ 0.20.

The concentration of IL-6 and IL-10 was determined by ELISA from the four groups of four mice and single-cell suspensions were prepared. After lysing red blood cells with Tris-NH_4_Cl, splenocytes (5 × 10^6 ^cells in 200 μl) were cultured at 37°C with E1+HVR1 recombination antigen (20 μg/ml)or LPS(1 μg/ml) in vitro, using the ELISA assay to measure the titer of IL-6 and IL-10. Results are shown as the mean value obtained for duplicate wells.

### 2.3. ELISPOT assay

Specific antibody secreting cells of E1 and HVR1 were quantified using an ELISPOT kit (Mabtech AB, Nacka Strand, Sweden). Multi Screen 96-well filtration plates MAIP S4510 (Millipore, USA, Masarzsa) were coated with 100 μl of a 50 μg/ml pBVIL6/E1 or pBVIL1/GST1+8 HVR1 dilution and 15 μg/ml anti-mouse IgG antibody dilution overnight at 4°C. Samples were then washed with ELISPOT coating buffer and blocked for 2 h with 200 μl RPMI1640 containing 10% Fetal bovine serum. After clearing red blood cells with Tris-NH_4_Cl, splenocytes (1 × 10^6^/well) were cultured for 24 h in RPMI-1640 alone (negative control). After removing cells and washing with wash buffer (PBS, 0.1% Tween 20), 100 μl of 1 μg/ml biotinylated anti-IgG in PBS containing 0.5% fetal bovine serum dilution was added and incubated for 1 h at room temperature. After washing, BCIP/NBT-plus substrate solution was filtered by passing through a 0.45 μm filter and added (100 μl/well) until the spots appeared at room temperature. After rewashing, spots were then counted using an immunospot image analyzer. Results are shown as the mean value obtained for duplicate wells.

### 2.4. Flow cytometric analysis following surface staining of splenocytes

Two weeks after the final immunization, spleens were harvested from four groups of four mice and single-cell suspensions were prepared. After lysing red blood cells with Tris-NH_4_Cl, splenocytes (5 × 10^5 ^cells in 200 ml) were cultured at 37°C with monoclonal antibodies directed against CD19, CD27 and CD38, namely anti-CD19-APC, anti-CD27-PE and anti-CD38-FITC(1 μl/well; Bioscience) as positive controls. The normal mice and anti-human CD19-APC served as negative controls. After 30 min, cells were washed and analyzed on a FACS Calibur flow cytometer (Becton Dickinson Immunocytometry Systems). For all flow cytometric analyses, the data (n = 4) are presented as means ± SD.

### 2.5. Immunoprecipitation

The virus capture activity of antisera was assessed by the modified method for immune complex analysis of HCV in patient sera. 100 microliters of 12 diluted patients' sera were centrifuged at 14 000 g for 15 minutes, and the supernatants were separately mixed with 5 μl of the poly anti-HVR1 IgG, which were purified from the immunization rabbits' serum with protein G sepharose. The mixtures were incubated for 4 h at 4°C, and then 5 μl of 2 mg/ml goat anti-rabbit IgG(Sigma)was added. After a second incubation period of 1.5 h at 4°C, the mixture were centrifuged at 2800 rpm for 15 min and separated into supernatants and pellets. To examine the capture activity of the anti-sera semiquantitatively, both the supernatant and the pellet were tested for HCV RNA by enzyme immunology assay combined RT-PCR(established by this laboratory). This method will be described next.

### 2.6. RT-PCR

RNA was extracted with Trizol(Life Technologies). A RT and a two-step PCR assay with nested primers were performed to detect the 5'-untranslated region of HCV RNA. The external pair of primers were F1 5'CG CTC *GAG *TTT GCC GGC GTT GAC G 3'and R1 5'CG *TCT *AGA AGT CCT GTT GAT GTG CC-3'. The sequence of the internal pair of primers were F2 5'-CG *CTC *GAG TTT GCC GGA GTT GAT G-3'and R2 5'-CG *TCT *AGA GGT CCT GTT GAT ATG CC-3'. In a 20 μl RT reaction system, 50 pmol of each external primer and 200 μmol/L dNTP were added. After denaturation for 5 minutes at 70°C, the mixture was bathed in ice immediately for 2 minutes. Then, 2U AMV (Promegar)was added, and the RT reaction was performed at 42°C for 1 hour. In the first step of PCR, the external pair of primer was used, and the cDNA was amplified in a 23 μl reaction mixture with 300 cycles of 30 s at 94°C, 30 s at 55°C and 30 s at 72°C each, followed by an extension for 5 minutes at 72°C at the end. In the second step, the internal pair of primer was used and 32 cycles were performed with each cycle of 30 s at 94°C, 30 s at 55°C and 30 s at 72°C, followed by an extension of 5 minutes at 72°C.

### Statistical analysis

Statistical analyses were performed using SAS 8.0 software. Results are presented as means ± standard errors. The statistical significance of the differences between the means of the experimental groups was tested by the Student t test for unpaired data. A difference was considered statistically significant when P < 0.05.

## 3. Results

### 3.1. Combined immunization with pUCpGs10 and Al (OH)_3_: the aluminum-induced humoral immune reaction

**Figure 1:**
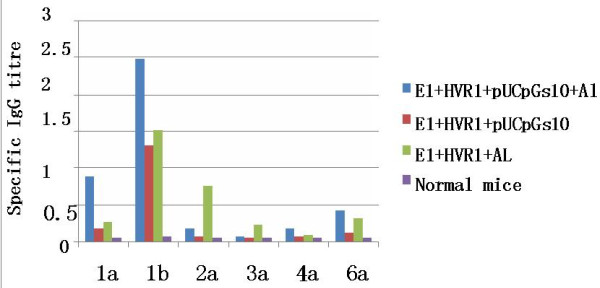
**Serum anti-E1a, E1b, E2a, E3a, E4a and E6a IgG responses following immunization with E1+HVR1 with pUCpGs10 and aluminium**. Five mice per group were immunized i.m. twice with a 4 week interval. The mice were bled 2 weeks after the third immunization to collect sera for the ELISA. The data are presented as mean serum IgG titres ± SD.

Differences between the four groups in terms of the induction of antigen-specific serum antibodies were strongly indicated since the mean titers of the four groups were 1:51200, 1:9051, 1:18102, 1:6400. To induce B-cell responses *in vivo*, mice were immunized (i/m) with the HCV polyprotein and different adjuvants. We found that the HCV polyprotein alone induces very low antigen-specific serum antibodies. In contrast, mixing the HCV polyprotein with the pUCpGs10 adsorbed to Al(OH)_3 _gel adjuvant induced the highest antibody responses. To determine whether differential specific responses were induced to each genotype component of the polyprotein, the serum antibody responses against each of the peptides (E1a, E1b, E2a, E3a, E4a and E6a) were measured [3] in the group immunized with Al (OH)_3_. These results demonstrate that serum antibody titers were greatest against the E1b and E1a peptides (Figure 1), where as the serum antibody responses were relatively low against the E3a and E4a, amounting to two orders of magnitude lower than anti-E2a and E6a titers. This data also conceived that, with majority of the antibody response being against the 1a and 1b genotypes for the folded conformation of the recombination polyprotein, although the expressed and purified E1 polyprotein was immunogenic in mice adjuvanted with pUCpGs10 and Al (OH)_3_, the response against each of the genotypes was different. The genotype 1a and 1b are most popular in China (40%, 16%). These results indicated that Al(OH)_3 _and pUCpGs10 plasmid combination stimulated a robust and long-term humoral immune response and also showed a synergistic effect. This is consistent with other reports that combination of pUCpGs10 with conventional adjuvants, such as Quil A [36], liposome [37], MPL and IFA/CFA [38], elicit synergistic antibody responses in mice.

### 3.2. Combined immunization with pUCpGs10 and Al(OH)_3 _induces the largest population of specific antibody secreting cells as assessed by the ELISPOT assay

**Figure 2:**
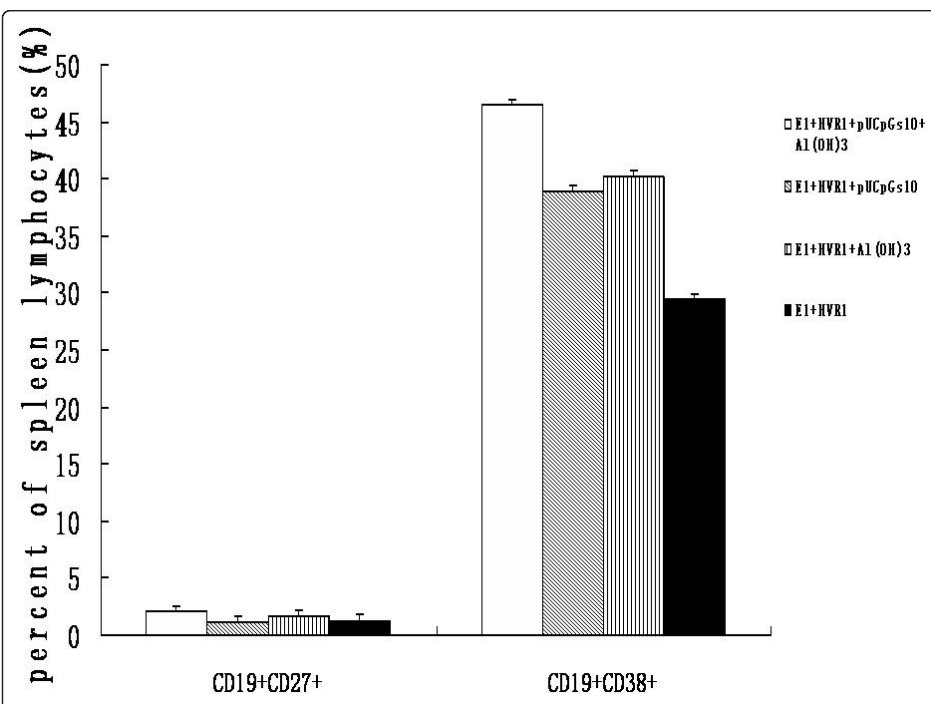
**The result of ELISPOT, FCM and ELISA of the spleen lymphocyte in BALB/c mice (mean ± SD)**. The application of aluminium have statistical significance of the population of the CD19^+^CD27^+ ^B cell and CD27^+^CD38^+^cell. (P < 0.01, P < 0.0001). The population of the CD19^+^CD38^+ ^B cell of Alum+CpG to the PBS group have statistical significance.(P < 0.05). The concentration of IL-6, IL-10 in cultivated supernatant of the splenic lymphocytes of the BALB/c mice after the incentivation of HCV antigen in vitro(mean ± SD)The concentration of IL-6 and IL-10 was determined by ELISA from the four groups of four mice and single-cell suspensions were prepared. After lysing red blood cells with Tris-NH_4_Cl, splenocytes (5 × 10^6 ^cells in 200 ml) were cultured at 37°C withE1+HVR1 recombination antigen (20 μg/ml)or LPS(1 μg/ml)in vitro, using the ELISA assay to measure the titer of IL-6 and IL-10. Results are shown as the mean value obtained for duplicate wells.

As a measure of B-cell responses, IgG-secreting responses in the spleen were measured after immunization with HCV polyprotein and adjuvants. In order to distinguish which adjuvant with the polyprotein were the most immunogenic, Tris-NH_4_Cl was used to isolate lymphocytes by the red cells. The total ASC (antigen secreting cell), which is also called functional plasma cell, contain antigen specific ASC and unspecific ASC. The population of the specific ASCs were 66.13 ± 26.66, 40.25 ± 22.22, 62.88 ± 17.06, 24.13 ± 14.90 SFU/10^6^; the population of total ASC were 111.25 ± 44.77, 73.00 ± 50.58, 125.75 ± 27.36, 42.50 ± 38.54 SFU/10^6^. Immunization with pUCpGs10 and aluminum induced the highest frequency of both IgG-secreting cells and total antigen secreting cells, while immunization with the HCV polyprotein alone induced the lowest frequency of IgG-secreting cells and total antigen secreting cells (Figure 2). The result show that aluminum can markedly increase the population of specific ASC (P < 0.01, F = 9.93) and total ASC (P < 0.05, F = 5.58) (Table 1). The proportion of specific ASC to total ASC were 59.4%, 55.14%, 50% and 56.76% (P < 0.05). This data demonstrates that CpG may increase the specific ASC to total ASC ratio.

**Table 1:** IgG-secreting cells and total antigen secreting cells produced by immunization with pUCpGs10 and aluminium.

Group	ELISPOT assay(SFU/10^6^)		FACS(%)		ELISA		
	Specific ASC	Total ASC	CD19^+^ CD27^+^	CD19^+^ CD38^+^	IL-6(pg/ml)	IL-10(pg/ml)	IgG titer
CpG+Alum	66.13 ± 26.66	111.25 ± 44.77	2.06 ± 0.34	46.42 ± 2.21	31.14 ± 34.57	249.75 ± 175.35	1:51200
CpG	40.25 ± 22.22	73 ± 50.58	1.15 ± 0.08	40.17 ± 6.40	15.74 ± 12.68	34.38 ± 2.29	1:9051
Alum	62.88 ± 17.06	125.75 ± 27.36	1.72 ± 0.49	38.91 ± 11.52	113.97 ± 101.98	241.50 ± 127.99	1:18102
PBS	24.13 ± 14.90	42.50 ± 38.54	1.29 ± 0.78	29.47 ± 8.49	5.88 ± .58	35.25 ± 9.36	1:6400

### 3.3. Combined immunization with pUCpGs10 and Al(OH)_3 _adjuvants induce different ratios of CD19^+^CD27^+^and CD19^+^CD38^+ ^B lymphocytes

**Figure 3:**
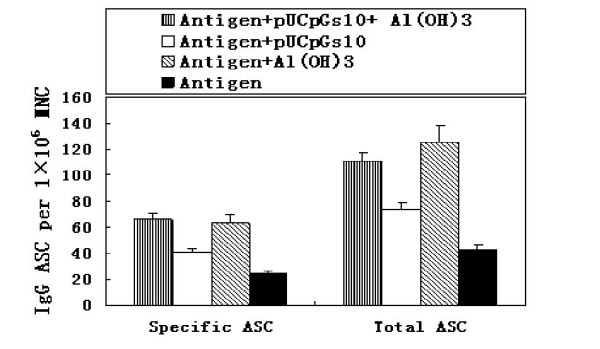
**(a-b). Houmal immunity responses by CD19^+^CD27^+ ^and CD19^+^CD38^+ ^B cells against polyprotein with different adjuvants measured by flow cytometry**. Mice were immunized three times with the HCVE1+HVR1 polyprotein and splenocytes were isolated. The surface of cells stained for CD19(APC), CD27(PE) or CD38 (FITC). The data are presented as the mean percentage ± SD of CD19 positive cells of total CD27^+ ^or CD38^+ ^cells of spleens of four groups from four mice. The proportion of CD19^+ ^CD27^+ ^spleen lymphocyte of the four groups are 2.06 ± 0.34, 1.15 ± 0.08, 1.72 ± 0.49, 1.29 ± 0.78(a); The proportion of CD19^+ ^CD38^+ ^spleen lymphocyte of the four groups are 46.42 ± 2.21, 40.17 ± 6.40, 38.91 ± 11.52, 29.47 ± 8.49(b);

The FCM revealed the ratio of different kinds of splenic lymphocytes. The ratio of CD19^+^CD27^+ ^B cells of the four groups were 2.06 ± 0.34, 1.15 ± 0.08, 1.72 ± 0.49, 1.29 ± 0.78(a); The ratio of CD19^+^CD38^+ ^B cells of the four groups are 46.42 ± 2.21, 40.17 ± 6.4, 38.91 ± 11.52, 29.47 ± 8.49(b)(Figure 2). The results show that the aluminum adjuvant can increase the ratio of CD19^+^CD27^+^(P < 0.05, F = 5.58) cells (Figure 3). However, the addition of Al (OH)_3 _to E1 + HVR1 did not enhance the ratio of CD19^+^CD38^+ ^cells further. The ratio of CD19^+^CD38^+ ^cells with the combination of aluminum and pUCpGs10 adjuvant is much higher than that of the control group(without adjuvant)(P < 0.01).

### 3.4. Concentrations of IL-6 and IL-10 in supernatant of cultured splenic lymphocytes in vitro

After using the ELISA assay to detect the concentration of cytokines in the supernatants of cultured splenic lymphocytes from the four groups (Figure 2), It was observed that the IL-6 and IL-10 concentrations after the aluminum adjuvanted and adjuvanted with a combination of aluminum and pUCpGs10 were markedly higher than those of the other groups(P < 0.05). And there are no differences between the LPS applied and control groups (P > 0.05). So it can be excluded that the impact of LPS mixed with the recombinant HCV polyprotein.

### 3.5. Poly anti-HVR1 IgG Immunoprecipitated with 12 patients' serum

**Figure 4:**
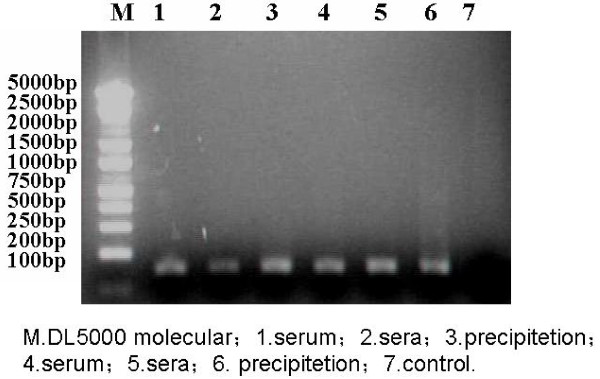
**HCV RNA could be detected both in the supernatant and in the precipitate(10/12)**. Both the supernatant and the pellet were tested for HCV RNA by enzyme immunology assay combined RT-PCR. The result show that HCV particles in one sample could be semi-precipitated by antiserum.

The result showed that HCV particles in one sample could be semi-precipitated by antiserum, that is, HCV RNA could be detected both in the supernatant and in the precipitate(10/12)(Figure 4).

## Discussion

Given that there are no effective vaccines currently available for HCV infection, an effective and safe immunization strategy eliciting the HCV-specific humoral response, e.g. the induction of antibody and effective B-cell response is especially important. Accordingly, this research used the recombinant HCV polyprotein adjuvanted with pUCpGs10 and aluminum hydroxide. It has been reported that inclusion of CPG7909 is associated with a greater proliferative response of PBMC to HBsAg at all time points following initial vaccination [39].

The present work suggests that aluminum can significantly enhance the humoral immune response by production of high levels of antigen specific antibodies. Moreover, the combination of pUCpGs10 with aluminum hydroxide can reinforce this effect to a level, much stronger than pUCpGs10 alone when assessed by ELISA assay. This study prompted us to assume that one immunological enhancement mechanism of the aluminum adjuvant is that it can conjugate the soluble antigens as particle format, a presentation ideally suited for internalization by APCs. It is known that the B lymphocyte's response to Al (OH)_3 _adjuvant is associated with the genesis of a memory immunologic response leading to long-term immunoprotection [21]. Therefore Al(OH)_3 _can promote a superior humoral immune response compared to other adjuvants.

Based on this knowledge, the mechanisms of humoral immunity enhancement by the pUCpGs10 and aluminum adjuvant can be studied. These results show that aluminum increases the populations of specific ASCs and the total ASCs, and when combined with pUCpGs10. Also, the ratio of specific ASC to total ASC can be increased as measured by the ELISPOT method. An analysis of the flow cytometric data showed that pUCpGs10 increases the performance of memory B cells and aluminum adjuvant likely enhanced this response. A similar enhancement was observed when immature B cells were activated by the pUCpGs10 combined with aluminum hydroxide. It can be predicted that the aluminum adjuvant is more potent than pUCpGs10 in enhancing the quality of the specific activated immature B cells, this will be studied in the future work. So, combining pUCpGs10 and aluminum with recombinant HCV polyprotein produces only moderate inflammation, but have the best effect through the most population of antigen-specific plasma cell, memory cell and highest titer of the specific antibody.

It can therefore to conclude that aluminum plays an important part in achieving humoral immune responses and persistent stimulation by pUCpGs10 adjuvants of the production of memory B cells. From these results, it is conceived that if the plasmid pUCpGs10 was applied alone, it would be degraded rapidly, would only promotes short term stimulation of memory B cells from the result of the further research in future. However, the memory B cells proliferate and differentiate into the specific ASC after receiving stimulation by antigen in the secondary immune response. The level of antibody and its persistence are related to the level of humoral immunity, and the population of specific ASCs directly correlated with the level of the antibody titer. The aluminum hydroxide adjuvant mainly enhances the level of specific ASCs and specific antibody titer, and its effect is much stronger than that of pUCpGs10. The pUCpGs10 stimulates innate immunity and decreases the population of non-specific ASCs, thus increasing immune efficiency. Furthermore, the addition of pUCpGs10 does not evoke an enhanced humoral immune reaction, but rather inhibits the non-specific immune response.

The Th2 type cytokines IL-6 and IL-10 promote the effectiveness of B cells at many levels. IL-6 stimulates B cell differentiation and enhances the secretion of Ig and the secondary immune response. IL-10 is an important Th2 cytokine to inhibit the antiviral immunity and attend the inflammation. The aluminum can enhance the concentration of IL-10. In this study, the FCM analyses specifically show that the aluminum adjuvanted animals have stronger enhancement of memory B cell compared to the others. Only the combination of aluminum and CpG increase the effectiveness of plasma cells, while the aluminum or CpG alone are unable to initiate such a response. At the same time, the ELISPOT assay results confirm that CpG increases the proportion of specific ASCs, so each adjuvant has a different and synergistic effect on the humoral immune reaction in mice.

10 out of 12 viruses of HCV patients' can treat with these immunoglobins. Using the cross-reactive and recombinant polyprotein good respondance can be achieved and will be considered as the new propriate vaccine of HCV. The result of the cooperation with SMMU (the second military medical university) is that the neutralization activity is very good in 19 quasispecies of 6 genotypes.

## Conclusions

These results suggest that the combination of pUCpGs10 with aluminium adjuvant may have wide application and further studies are warranted. Relevant and further studies are now planned in this laboratory.

## Competing interests

The authors declare that they have no competing interests. In the past three years I have not received funding from an organization that may gain financially from the publication of this manuscript now. I do not hold any stocks or shares in an organization that may in any way gain or lose financially from the publication of this manuscript, neither now nor in the future. I am not currently applying for any patents relating to the content of the manuscript. I do not have any other financial competing interests.

## Authors' contributions

XGS and CXZ carried out the molecular genetic studies; GZB participated in the sequence alignment and drafted the manuscript. GHW and KC, carried out the immunoassays. ZHD and XQY participated in the sequence alignment. NZ and BSX participated in the design of the study and performed the statistical analysis. HQZ conceived of the study and participated in its design and coordination. All authors read and approved the final manuscript.
